# Flavonoids and antioxidant activity of rare and endangered fern: *Isoetes sinensis*

**DOI:** 10.1371/journal.pone.0232185

**Published:** 2020-05-12

**Authors:** Xin Wang, Guohua Ding, Baodong Liu, Quanxi Wang

**Affiliations:** 1 College of Life Science and Technology, Harbin Normal University, Harbin, China; 2 College of Life and Environmental Sciences, Shanghai Normal University, Shanghai, China; 3 Shanghai Key Laboratory of Plant Functional Genomics and Resources, Chinese Academy of Sciences, Shanghai Chenshan Botanical Garden, Shanghai, China; University of Delhi, INDIA

## Abstract

*Isoetes sinensis* Palmer is a critically endangered, first-class protected plant in China. Until now, researchers have primarily focused on the ultrastructure, phylogeny, and transcriptomes of the plant. However, flavonoid profiles and bioactivity of *I*. *sinensis* have not been extensively investigated. To develop the endangered *I*. *sinensis* for edible and medicinal purposes, flavonoid content, chemical constitution, and antioxidant activities were investigated in this study. Results revealed the following. 1) The total flavonoid content was determined as 10.74 ± 0.25 mg/g., 2) Antioxidant activities were stronger than most ferns, especially ABTS free radical scavenging activities. 3) Four flavones, containing apigenin, apigenin-7-glucuronide, acacetin-7-O-glcopyranoside, and homoplantageninisoetin; four flavonols, namely, isoetin, kaempferol-3-O-glucoside, quercetin-3-O-[6”-O-(3-hydroxy-3-methylglutaryl)-β-D-glucopyranoside], and limocitrin-Neo; one prodelphinidin (procyanidins;) and one nothofagin (dihydrochalcone) were tentatively identified in the mass spectrometry-DAD (254nm) chromatograms. This study was the first to report on flavonoid content and antioxidant activities of *I*. *sinensis*. Stronger antioxidant activity and flavonoid content suggests that the endangered *I*. *sinensis* is an important and potentially edible and medicinal plant.

## Introduction

Evidence based on epidemiological and pharmacological data has shown that flavonoids play an important role in preventing and managing modern diseases [1–5]. Total flavonoid content and antioxidant capacities have been the main focus of research on medicinal and food applications of natural phytochemicals [6–8].

*Isoetes* are considered rare living fossils. With the rapid development of the economy resulting in habitat degradation, wild populations have declined dramatically. Thus far, resource utilization of *Isoetes* has yet to be reported, especially for the specific species, *Isoetes sinensis* Palmer. *I*. *sinensis* was a common species in the wetlands of An’hui, Jiangxi, Jiangsu, and Zhejiang Provinces before 1980. In the last 20 years, this species has all but disappeared [9]. Due to this dramatic decline, *I*. *sinensis* has been considered a critically endangered, first-class protected plant in China since 1999 [10–12]. Fortunately, our lab has propagated tens of thousands of *I*. *sinensis* by way of creative spore propagation, which has allowed for the development of *Isoetes* and its continued use in present and future research.

As of today, the ultrastructure, transcriptome, numerous sequences, and functional genes of *I*. *sinensis* have been reported [12–16]. However, the flavonoids and antioxidant activity from *Isoetes* have been scantily studied. The goal of this study was to report on flavonoid content and antioxidant activities of *I*. *sinensis* for edible and medicinal development

## Materials and methods

### Plant materials

Plants were collected from “Fern Garden” in the Botanical Garden of Harbin Normal University. “Fern Garden” was a greenhouse which served for scientific research and teaching. After overcoming various reproductive difficulties, *Isoetes sinensis* was cultivated successfully in “Fern Garden” with proper temperature (18°C -35°C), luminous intensity (1500Lx-3000Lx) and relative humidity (35%-80%). *Isoetes sinensis* were cultivated,. So, no specific permissions were required for this location. Plants were identified by Prof. Liu who worked in Harbin Normal University and engaged in the reproductive development of ferns and the propagation of endangered ferns for 30 years in China. Voucher specimens were deposited in the College of Life Science & Technology at Harbin Normal University.

### Chemicals and reagents

Refer to our previous report [17] for an overview of the chemicals and reagents used in this study. Rutin (purity > 99.0%), 2,2-Diphenyl-1-picrylhydrazyl (DPPH), 2,2’-azinobis-(3-ethylbenzothiazoline-6-sulfonic acid) (ABTS), Nitrotetrazolium blue chloride (NBT), phenazine methosulfate (PMS), nicotinamide adenine dinucleotide (NADH), 5, 5’-dithiobis-(2-nitrobenzoic acid) (DTNB) and 2,4,6-tri-2-pyridyl-s-triazine (TPTZ) were purchased from Sigma Co. (Shanghai, China). Acetonitrile was purchased from Thermo Fisher Scientific (Shanghai, China).

### Preparation of plant extracts

Fresh plant materials were cleaned by distilled water and then dried under the outdoor shady conditions, finally, at roughly 75°C for 48 h in an electro- thermostatic blast oven (Bluepard Instruments CO., LTD, Shanghai, China). Materials were then powdered by the pulverizer and filtered through a 40-mesh screen. 1g dried sample was separately extracted with 25 mL of 60% ethanol for 2 h at 50°C in the polyscience. Ultrasound-assisted extraction was performed for 20 min. The extraction process was repeated twice. The mixture was then filtered via a vacuum suction filter pump and the extract solutions were collected. The extract solution was measured at 8 mL and was extracted twice with 8 mL petroleum ether, and then the residue solution was extracted with dichloromethane, ethyl acetate, and n-butanol, respectively. Each fraction was concentrated to dryness by evaporation on a rotary evaporator and dissolved with 1 mL ethanol, respectively. Before testing, the solutions were filtered through a 0.45-μm membrane (Millipore, Billerica, MA, USA). Samples were then prepared for HPLC analysis.

### Determination of total flavonoids content

Total flavonoid content was measured by a colorimetric assay. Rutin was used to draw a calibration curve [17]. The following formula was used:

$$Total\;flavonoid\;content\;\left(\%\right)=\left[\left(OD_{1}+OD_{2}+OD_{3}\right)/3‐A\right]/B*10/2*Volume/1000*100\%.$$

#### Antioxidant activity DPPH assay

1 mL 0.1 mM DPPH and extracts were mixed for 30 min. The optical density was measured and recorded at 517 nm. For the control group, 60% methanol was used. Experiments were performed in triplicate with similar results (RSD < 5.0%). DPPH free radical scavenging activity is determined with:

$$\left(\%\right)=\left(1‐A_{sample\;517}/A_{control\;517}\right)*100.$$

Please refer to [17] for an overview of the DPPH assay used in this study.

#### ABTS assay

150 μL extracts and 3 mL ABTS solutions with an optical density of ± 0.9 mixed for 6 min. The absorbance value was determined to be 734 nm. Experiments were performed in triplicate with similar results (RSD < 5.0%). ABTS free radical scavenging activity is determined with:

$$\left(\%\right)=\left(1‐A_{sample\;734}/A_{control\;734}\right)*100.$$

Please refer to [17] for an overview of the ABTS assay used in this study.

#### Superoxide anion (O^2-^) scavenging activity

1 mL NBT (150 μM), 1 mL NADH (468 μM), and 1 mL PMS (60 μM) were consecutively added to 1 mL the mixture of extracts and sodium phosphate buffer. After incubation for 5 min at 25°C, the optical density was determined to be 560 nm. For the control group, 60% methanol was used. Superoxide anion scavenging activity was determined by:

$$\left(O^{2‐}\right)\;scavenging\;activity\;\left(\%\right)=\left(1‐A_{sample\;560}/A_{control\;560}\right)*100.$$

Experiments were performed in triplicate with similar results (RSD < 5.0%). Please refer to [17] for an overview of the scavenging activity assay used in this study.

#### Reducing power assay

1 mL extracts, 2.5 mL phosphate buffer, and 2.5 mL potassium ferricyanide were mixed and then placed in a water bath at 50°C for 20 min. 2.5 mL TCA was added to terminate the reaction. After centrifuging for 10 min, 2.5 mL supernatant was added to 2 mL distilled water and 0.5 mL 0.1% ferric chloride. 2.5 mL supernatant was added to 3 mL distilled water as part of the control group. The optical density was recorded at 700 nm and reflected the reducing power. The experiments were performed in triplicate with similar results (RSD < 5.0%). Please refer to [17] for an overview of the reducing power assay used in this study.

#### FRAP assay

The FRAP reagent, which was made up with 10 mM TPTZ in 40 mM HCl solution and 20 mM FeCl_3_ in 250 mL acetate buffer, pH 3.6. 50 μL Extracts were mixed with the 1.5 mL FRAP reagent for 4 mins. The optical density of the mixture was recorded at 593 nm. The FRAP reagent was mixed with 50 μL distilled water as part of the control group. The experiments were performed in triplicate with similar results (RSD < 5.0%). The FRAP assay was used in this study as detailed in [17].

#### Flavonoids analysis of *I*. *sinensis* by HPLC-ESI-TOF-MS

Lastly, an Agilent 1100 HPLC system (Agilent Technologies, USA) was used to perform the chromatographic separation. The system was equipped with an abinary pump, amicrodegasser, Hi-performance well-plateauto sampler, thermostated column compartment, and diode-array detector (DAD). The UV Spectrum was recorded between 190–400 nm; the UV detector was set at 254 nm. SHISEIDO MG-C18 (1003.3 mm; i.d. 3.0 mm) column using a gradient elution [methanol (A)/ water (0.1% HCOOH)(B)] was chosen. All of the MS experiments were conducted on an Agilent 6220 Time-of-Flight mass spectrometry (TOF) equipped with an electrospray ionization (ESI) interface (Agilent Technologies, USA).

The gradient condition was 0–15 min at 15–45% A, 15–25 min at 45–55% A, and 25–35 min at 55–90% A. The column temperature was set at 25°C, the flow rate was kept at 0.4 mL/min, and the injection volume was 10 μL. Both the auxiliary and nebulizer gases consisted of nitrogen with a flow rate of 10 L/min. The MS analysis was performed in both positive and negative scan modes under the following operation parameters: the nebulizer pressure was set at 45 psi, the dry gas temperature was set at 350°C, and the voltage was set at 160 V. Full scan data acquisition and dependent scan event data acquisition were performed from m/z 100–1200.

## Results and discussion

### Total flavonoid content of *I*. *sinensis*

The concentration and total flavonoid content of *I*. *sinensis* were determined as 0.043 mg/ml, 10.74 ± 0.25 mg/g, respectively. Although far lower than most species of filicinae, a little higher than that of *selaginella* and *equisetums* [18], and higher than the total flavonoid content of bryophytes [19]. Primitive plant groups might have lower flavonoid contents than their modern counterparts.

### Antioxidant activity

**Fig 1:**
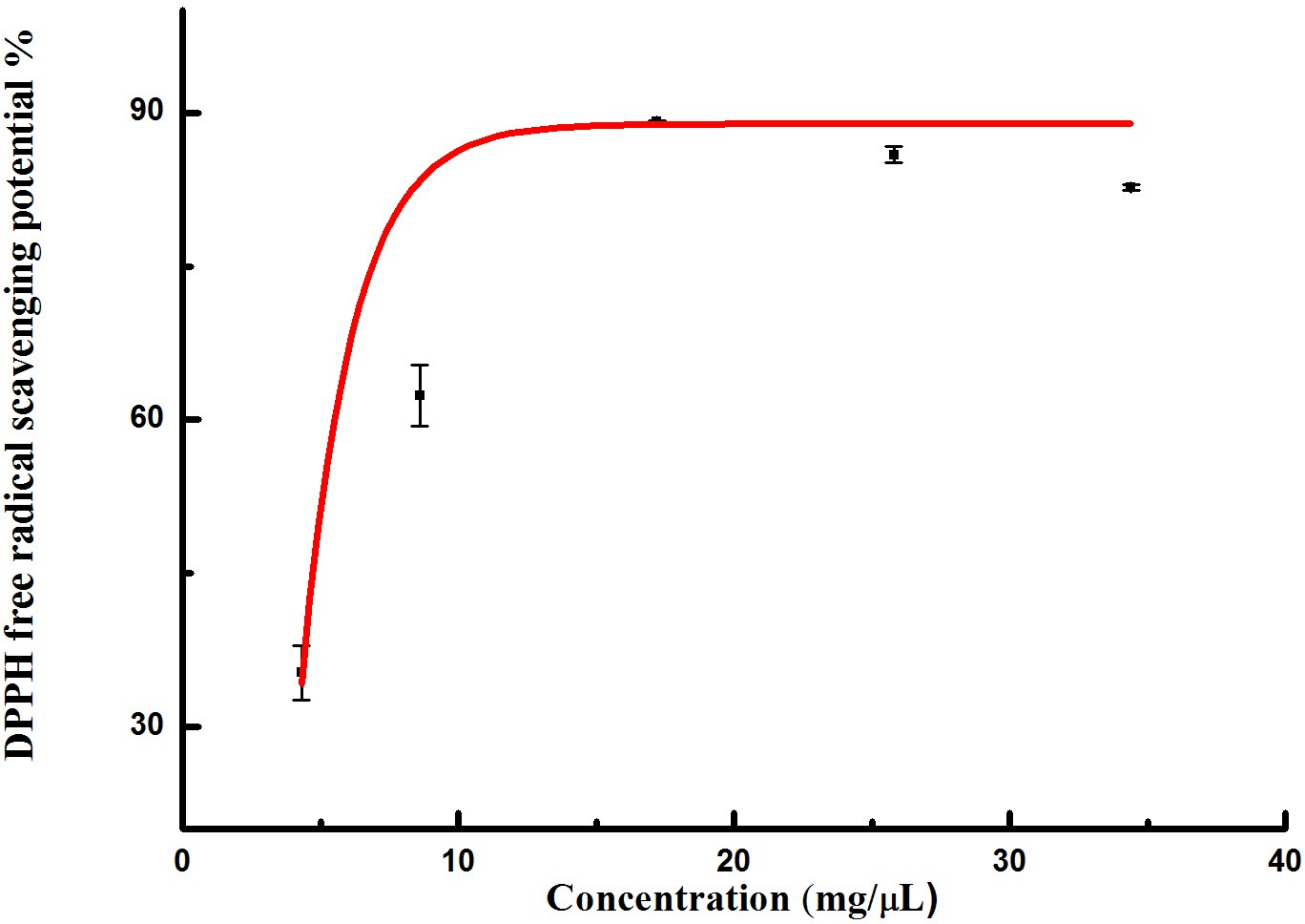
DPPH free radical scavenging activity observed in extracts from *I*. *sinensis*.

With the increasing of the concentration, the DPPH free radical scavenging potential observably increased (Fig 1). Roughly 111.9 μL of the extracts could scavenge about 50% of the free radicals. The IC_50_ of DPPH scavenging activity was recorded as 4.8 mg/μL, 10 mg/μL extracts could scavenge 100% DPPH free radicals.

**Fig 2:**
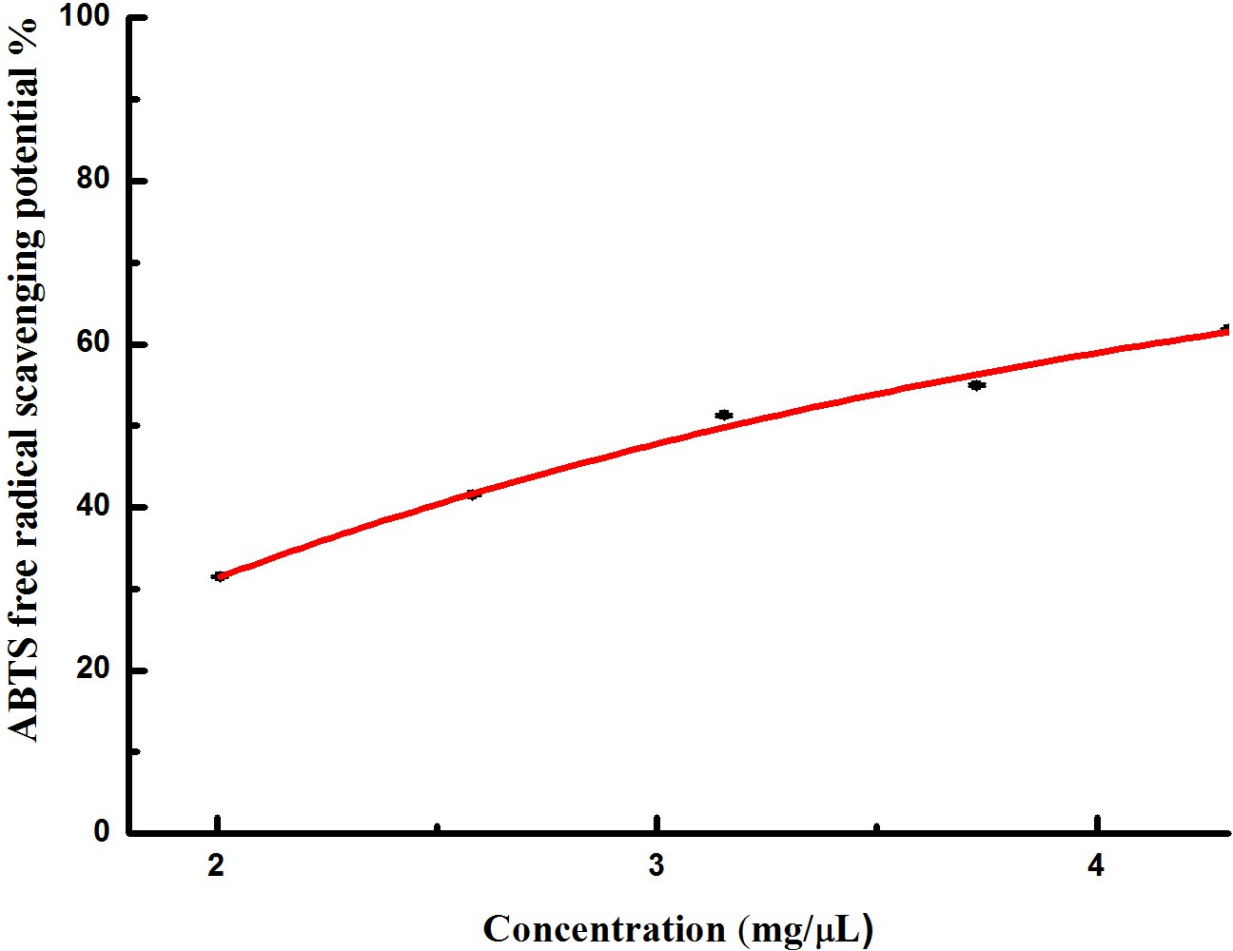
ABTS free radical scavenging activity observed in extracts from *I*. *sinensis*.

ABTS free radical scavenging potential noticeably increased with the increasing of the concentration (Fig 2). Roughly 11 μL of the extracts could scavenge about 50% of the free radicals. The IC_50_ of ABTS scavenging activity was recorded as 3.2 mg/μL.

**Fig 3:**
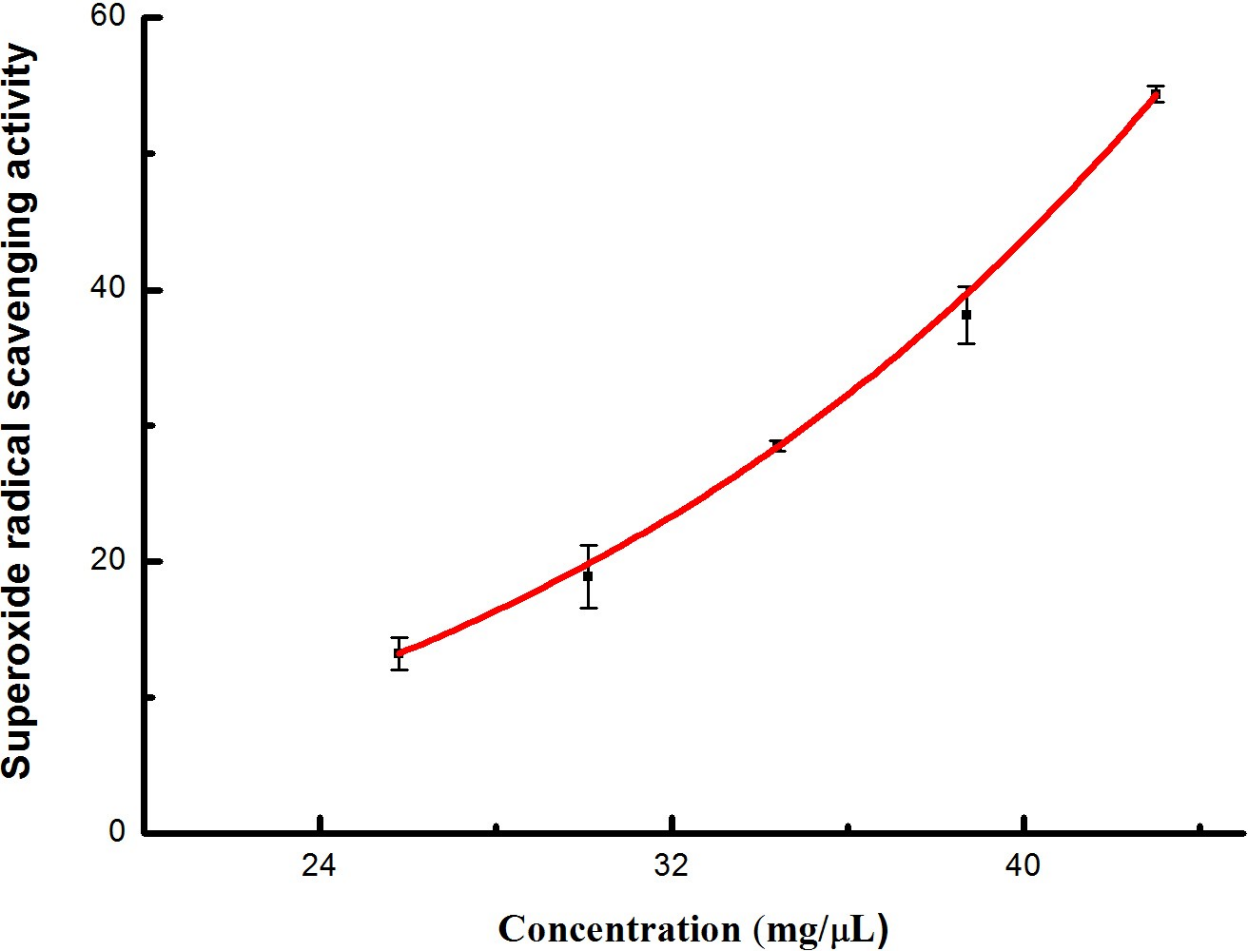
Superoxide radical scavenging activity observed in extracts from *I*. *sinensis*.

Superoxide radicals scavenging potential noticeably increased was relative to the increasing concentration (Fig 3). Roughly 991.5 μL of the extracts could scavenge about 50% of the free radicals. The IC_50_ of superoxide radicals scavenging activity was recorded as 42.6 mg/μL.

**Fig 4:**
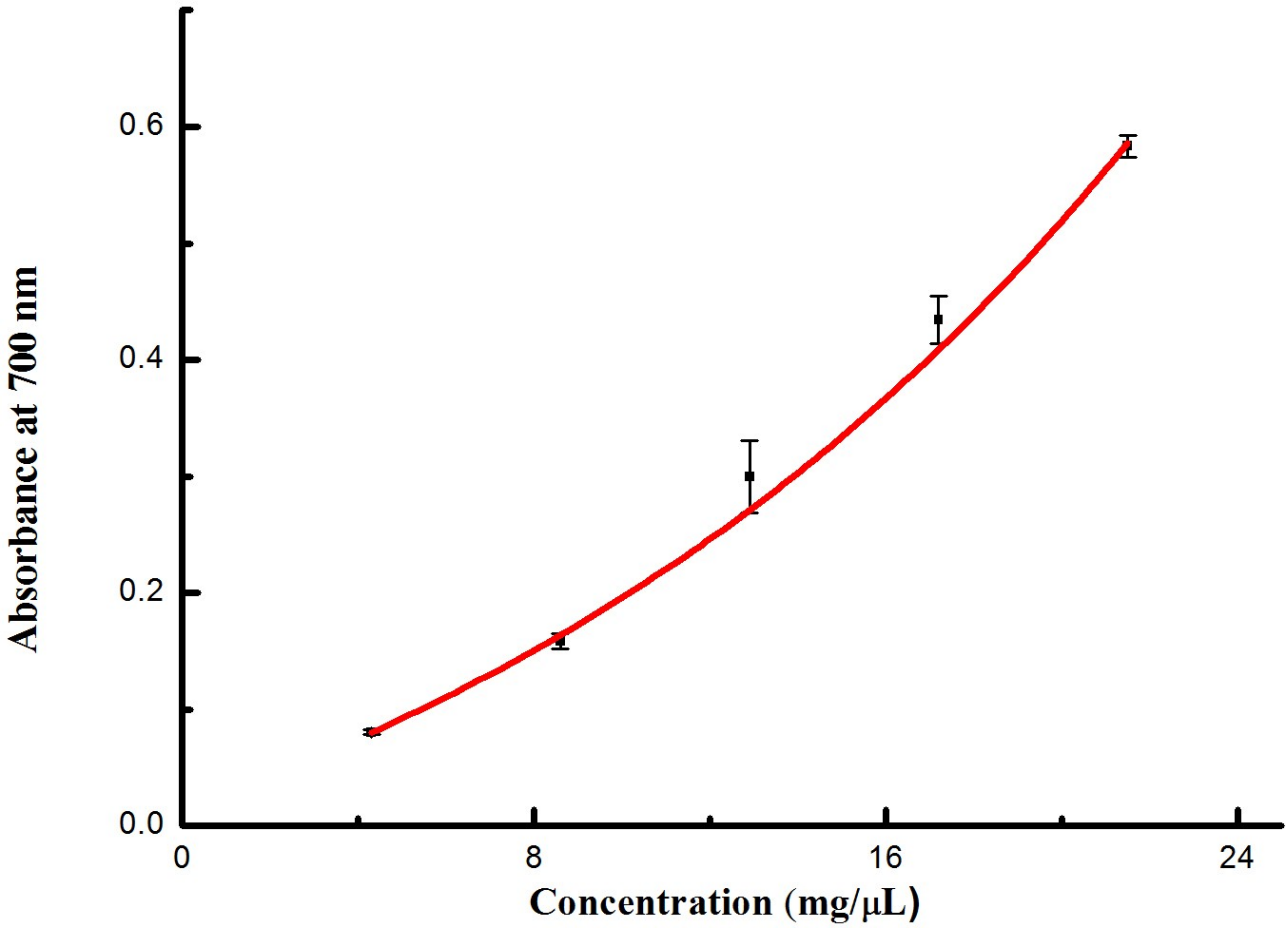
The reducing power observed in extracts from *I*. *sinensis*.

**Fig 5:**
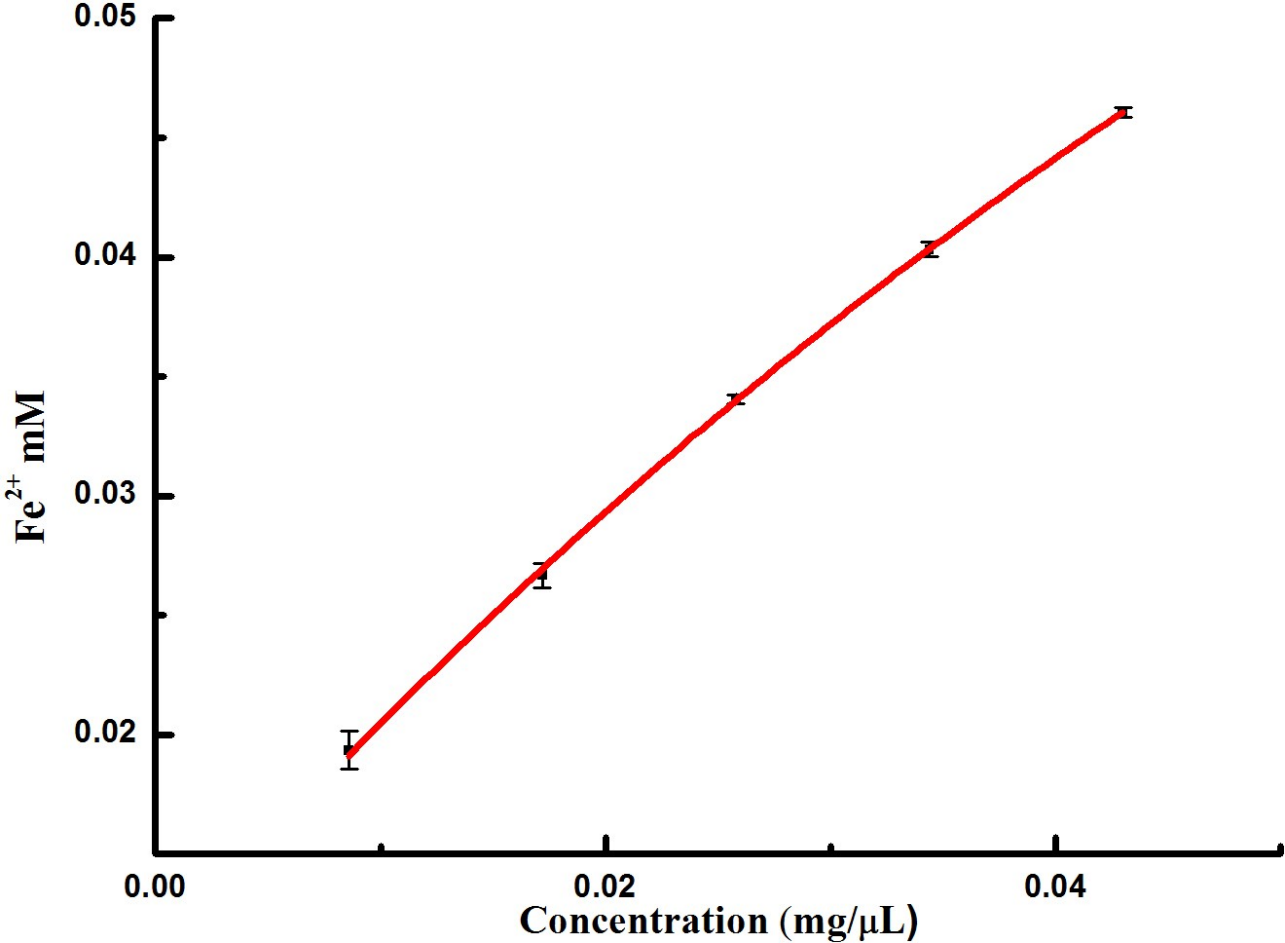
Antioxidant power by FRAP assay observed in extracts from *I*. *sinensis*.

The results from the FRAP and reducing power assays illustrated that the extracts from *I*. *sinensis* possessed antioxidant and reductive activity of Fe^3+^ (Figs 4 & 5). With increasing volume, the activity clearly improved.

Based on the results noted above, the antioxidant activities of *I*. *sinensis* were obviously stronger than most reported ferns and bryophytes, especially in terms of ABTS-free radical scavenging activities [19, 20]. This showed that *I*. *sinensis* had clear medicinal implications. *I*. *debii* Sinha was commonly used in Indian cuisines [21] and roasted rhizomes are used in cough and cold medication [22]. With stronger antioxidant activity than other species of its kind, *I*. *sinensis* has potentially important applications in both food and medicine.

### Flavonoids analysis of *I*. *sinensis* by HPLC-ESI-QTOF-MS

**Fig 6:**
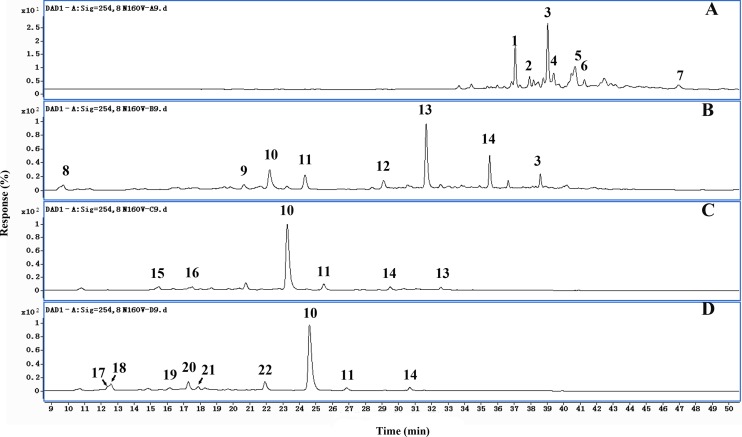
DAD (254nm) chromatograms of flavonoid extracts from *I*. *sinensis*: a) Petroleum ether fraction; b) dichloromethane fraction; c) ethyl acetate fraction; and d) n-butanol fraction.

HPLC-ESI-QTOF-MS was utilized in the qualitative analysis of flavonoid of *I*. *sinensis* (Table 1). In comparison with the known chromatograms and mass spectral data in literature, a total of 22 peaks were tentatively identified from 4 extracts: petroleum ether fraction (A), dichloromethane fraction (B), ethyl acetate fraction (C), and n-butanol fraction (D) (Fig 6).

**Table 1:** Analysis of *I*. *sinensis* by HPLC-ESI-QTOF-MS. a) petroleum ether fraction; b) dichloromethane fraction; c) ethyl acetate fraction; and d) n-butanol fraction.

No.	RT	Compound type	Formula	MW	Observed mass[M-H]-	Calculated mass[M-H]-	Mass error (ppm)	UV λ max/nm	Identification	Part	Ref
1.	37.02	benzoic acid	C15H22O2	234.1615	233.1542	233.1547	2.02	245	4-Octylbenzoic acid	A	[17]
2.	37.91	fattyacid	C16H28O3	268.2039	267.1966	267.1966	-0.04	250	3(ζ)-Hydroxy-hexadeca-4(E),6(Z)-dienoic acid	A	[31]
3.	38.72	fattyacid	C18H30O3	294.2197	293.2124	293.2122	-0.63	250, 295	9-Hydroxy-10E-octadecen-12-ynoic acid	A	[32]
4.	39.43	fattyacid	C18H32O3	296.2349	295.2276	295.2279	0.76	255	9-,13-Hydroxyoctadecadienoic acid	A	[33]
5.	40.75	fattyacid	C20H34O2	306.2562	305.2489	305.2486	-1.05	245	Dihomolinolenic	A	[34]
6.	41.31	ketone	C30H48O5	488.3505	487.3432	487.3429	-0.57	255, 275, 320	Ganodermanontetrol	A	[35]
7.	47.03	fattyacid	C20H34O2	306.2562	305.2489	305.2486	-1.05	245	8,11,14-Eicosatrienoic acid	A	[36]
8.	9.72	benzoic acid	C7H6O3	138.0317	137.0244	137.0244	0.23	258	P-Hydroxybenzoic acid	BB	[37]
9.	20.67	benzoic acid	C14H12O3	228.0781	227.0708	227.0714	2.35	238, 305, 320	4-(Phenoxymethyl)benzoic acid	B	[38]
10.	22.20	flavone	C21H18O11	446.0853	445.078	445.0776	-0.85	240, 268, 340	Apigenin-7-glucuronide	B	[31]
11.	24.35	flavone	C22H22O10	446.1208	445.1135	445.114	1.08	240, 260, 335	Acacetin-7-O-glcopyranoside	B	[39]
12.	28.48	flavonol	C15H10O6	286.0473	285.04	285.0405	1.62	252, 270, 352	Isoetin	B	[40]
13.	31.69	flavone	C15H10O5	270.0523	269.0451	269.0455	1.79	238, 269, 340	Apigenin	B	[41]
14.	35.57	coumarin	C16H12O5	284.0681	283.0608	283.0612	1.45	240, 268, 340	Tomenin	B	[42]
15.	15.50	dihydrochalcone	C21H24O10	436.1376	435.1303	435.1297	-1.46	238, 310	Nothofagin	C	[43]
16.	17.52	flavonol	C21H20O11	448.1007	447.0934	447.0933	-0.28	260, 340	Kaempferol-3-O-glucoside	C	[44]
17.	12.40	lactic acid	C9H10O3	166.0634	165.0561	165.0557	-2.35	240, 300, 320	Phenyllactic acid	D	[45]
18.	12.64	coumarin	C16H18O9	354.0955	353.0882	353.0878	-1.05	245, 295, 326	Scopolin	D	[46]
19.	16.12	procyanidins	C27H30O15	594.1591	593.1518	593.1512	-1.01	245, 272, 330	Prodelphinidin	D	[47]
20.	17.27	flavonol	C27H28O16	608.1388	607.1315	607.1305	-1.75	240, 268, 320	Quercetin-3-O-[6″-O-(3-hydroxy-3-methylglutaryl)-β-d-glucopyranoside]	D	[48]
21.	17.89	flavonol	C29H34O17	654.1795	653.1722	653.1723	0.16	245, 265, 325	Limocitrin-neo	D	[49]
22.	21.91	flavone	C21H18O12	462.0808	461.0735	461.0725	-2.1	255, 268, 350	Homoplantagenin	D	[50]

Four flavones contain apigenin, apigenin-7-glucuronide, acacetin-7-O-glcopyranoside, and homoplantageninisoetin; four flavonols, namely, isoetin, kaempferol-3-O-glucoside, quercetin-3-O-[6”-O-(3-hydroxy-3-methylglutaryl)-β-D-glucopyranoside], and limocitrin-Neo; one prodelphinidin (procyanidins); and one nothofagin (dihydrochalcone) were tentatively identified in the mass spectrometry-DAD (254nm) chromatograms of extracts from *I*. *sinensis*. Apigenin was an edible natural flavonoid found in several dietary plant foods such as vegetables and fruits and showed potential antioxidant, anti-inflammatory and anticancer properties [23]. Hepatoprotective effects of kaempferol-3-O-glucoside had been proved [24]. Prolonged diuretic and saluretic effect of nothofagin had been proved [25]. It was inferred that *I*. *sinensis* was potential edible and medical plant.

Flavonoid content of Isoetaceae was poorly known but appears to contain mainly flavone, apigenin, luteolin, chrysoeriol, selgin, tricin, and isoetin (as the 5'-glucoside), which had been reported in other species of *Isoetes* [26]. Expectisoetin, apigenin procyanidins and dihydrochalcone might exist in *I*. *sinensis* as well. Flavones and flavonols were the main flavonoids in most species from filicinae [18]. Flavones, flavonols, flavanones, aurones, and dihydrochalcones were the main flavonoids in bryophytes [27]. The dihydrochalcones observed in this study suggested that Isoetaceae and bryophytes may be phylogenetically similar.

Effects of various ecological factors on the secondary metabolite profile have also been observed in angiospermae [28]. In a previous study [18, 20], a similar impact of ecological factors on the flavonoids found in the same species. The lower flavonoid content of aquatic ferns was attributed to specific environmental factors, which typically require no need to produce flavonoids in self-defense. *Pilularia globulifera*, a marsileaceous fern, was previously reported to accumulate quercetin and kaempferol glycosides [29], which was similar to the content found within *I*. *sinensis*. An aquatic macrophyte, *Stratiotes aloides*, accumulated luteolin and chrysoeriol glycosides [30], which were different from *I*. *sinensis*. Thus, it was speculated that the phytogroup was also influencing factors on the secondary metabolite profile, in addition to the ecological factors.

## Conclusion

This study is the first to report on the phytochemistry and biological activities of *I*. *sinensis*. The results showed that *I*. *sinensis* was with stronger antioxidant activity than some fern and bryophytes and higher flavonoid content (10.74 ± 0.25mg/g), So the endangered *I*. *sinensis* should be an important and potentially edible and medicinal plant.
